# Transfection With Plasmid Causing Stable Expression of a Foreign Gene Affects General Proteome Pattern in *Giardia lamblia* Trophozoites

**DOI:** 10.3389/fcimb.2020.602756

**Published:** 2020-12-18

**Authors:** Manfred Heller, Sophie Braga, Norbert Müller, Joachim Müller

**Affiliations:** ^1^ Proteomics & Mass Spectrometry Core Facility, Department for BioMedical Research (DBMR), University of Bern, Bern, Switzerland; ^2^ Department of Infectious Diseases and Pathobiology, Vetsuisse Faculty, Institute of Parasitology, University of Bern, Bern, Switzerland

**Keywords:** model organisms, reverse genetics, untargeted proteomics, experimental proteomics, controls and standards

## Abstract

*Giardia lamblia* is an important causative agent of persistent diarrhea in humans, domestic animals, and cattle. Basic research is usually performed with the strain WBC6 and includes genetic manipulations such as transfections. Here, we investigate how transfection with a plasmid causing stable expression of a foreign gene affects the whole proteome pattern. Using shotgun mass spectrometry, we compare the proteomes of untransfected trophozoites to trophozoites transfected with *Escherichia coli* glucuronidase A (GusA). Besides GusA, which is detected in the transfected trophozoites only, the proteomes of untransfected and transfected trophozoites differ by 132 differentially expressed proteins. In particular, transfection induces antigenic variation. Since transfection causing stable expression affects the proteome pattern, transfection experiments should take into account this effect. Due to a unique peptide panel, GusA is an example for a suitable internal standard for experiments involving transfected cells. Data are available *via* ProteomeXchange with identifier PXD022565.

## Introduction

The diplomonadid *Giardia lamblia* (syn. *G. duodenalis*, *G. intestinalis*), is an early diverging, anaerobic eukaryote (Müller and Müller, 2016; Cernikova et al., 2018) causing persistent diarrhea, especially in regions with low hygienic standards (Hemphill et al., 2019). After stomach passage, ingested cysts transform into trophozoites colonizing the duodenum. Hosts in good physical condition face the strongest symptoms of giardiasis 1 week post infection, and recover within two to three weeks. Rarely, the infection becomes chronical causing severe damage of the intestinal epithelium, which may result in the development of irritable bowel syndrome (Allain et al., 2017; Litleskare et al., 2018). Giardiasis can be regarded as a zoonosis since it occurs in humans as well as in other mammals (Thompson, 2004).

The largest part of laboratory research is based on the strain WBC6, cloned from an isolate obtained from a patient (with the initials W.B.) suffering from chronical giardiasis (Nash et al., 1985; Campbell and Faubert, 1994). WBC6 is amenable to reverse genetics *via* transfection with double-stranded RNA (Furfine and Wang, 1990) or DNA (Singer et al., 1998; Sun et al., 1998), respectively. The genome of WBC6 (Morrison et al., 2007) and of other strains have been sequenced so far (see www.giardiadb.org). One unique feature of the *Giardia* genome is the presence of more than hundred open reading frames encoding cysteine-rich surface proteins, the so-called “variant-specific surface proteins” (VSPs), the “cysteine-rich proteins”, and the “high cysteine membrane proteins” (HCMPs). In a recently published chromosome-scale reference genome, 133 genes encoding for VSPs, as well as other highly repetitive genes such as 184 never-in-mitosis gene a (NIMA) related kinases and 305 ankyrin-repeat proteins (formerly annotated as proteins 21.1), are distributed over all five chromosomes (Xu et al., 2020). VSPs are considered as the predominant surface antigens of ***G. lamblia*** trophozoites (Adam et al., 2010). According to a generally admitted hypothesis, one single trophozoite expresses only one VSP at any one time (Nash et al., 2001). The switching from one VSP to another is called “antigenic variation” (Nash, 2002). Antigenic variation depends on epigenetic and post-transcriptional mechanisms, as evidenced by various studies (Kulakova et al., 2006; Prucca et al., 2008) and reviewed elsewhere (Prucca et al., 2011; Lagunas-Rangel and Bermudez-Cruz, 2018).

Shotgun mass spectrometry proteome studies have revealed that WBC6 trophozoite populations and trophozoite populations from other strains express an impressive number of VSPs at the same time (Emery et al., 2014; Emery, Lacey et al., 2015a; Emery et al., 2015b). Moreover, strain-dependent antigenic variation occurs upon drug pressure, concomitantly to large changes in expression patterns of other proteins (Emery et al., 2018; Müller et al., 2019).

As for many other model organisms, reverse genetics based on transfection with suitable plasmids or RNA viruses leading to transient or stable overexpression of genes of interest constitute an important research tool for *Giardia* (Davis-Hayman and Nash, 2002). In current protocols, transfectants are selected *via* a resistance marker encoded by the transfected plasmid, in particular neomycin phosphotransferase (Sun et al., 1998) and puromycin acetyltransferase (Singer et al., 1998; Jiménez-García et al., 2008). Since exposure to drugs and selection of resistance causes marked changes in gene expression patterns, it is logical to ask which side effects the transfection procedure has on gene expression in the transfected cells, independently of the transfected gene. In one of the first studies addressing this topic on the transcriptional level, neomycin and puromycin selection affected the expression patterns of various genes (Su et al., 2007).

Based on these findings, we contend that stable transfection of *G. lamblia* based on puromycin selection induces changes in proteome patterns to the same extent as differences amongst strains from different genotypes and differences between drug resistant and susceptible strains of the same background. Using shotgun mass spectrometry, we compare the proteomes of *G. lamblia* WBC6 trophozoites transfected with a plasmid containing *Escherichia coli* glucuronidase A (GusA) and *Streptomyces alboniger* puromycin_N-acetyltransferase (Pac) as a resistance marker (Singer et al., 1998) with untransfected trophozoites. The GusA transfection plasmid has been used by our group as a standard control in previous experiments (Nillius et al., 2011; Müller et al., 2013) involving transgenic *Giardia*. GusA-transfected trophozoites are not affected in growth (Müller et al., 2009) and have a metabolomics profile similar to untransfected trophozoites (Müller et al., 2020b). Here, we investigate the impact of transfection on the proteome pattern with a major focus on antigenic variation. Furthermore, we ask the question whether the transfected transgenes, i.e. the resistance marker Pac, GusA, or both can be used to estimate the expression level of the transgene in comparison to “housekeeping” proteins.

## Materials and Methods

### Chemicals

If not otherwise stated, all biochemical reagents were from Sigma (St Louis, MO, USA). Puromycin was obtained from Invivogen (Toulouse, France).

### Axenic Culture, Harvest, and Storage of *Giardia lamblia* Trophozoites

Trophozoites from *G. lamblia* WBC6 were grown under anaerobic conditions in 10 ml culture tubes (Nunc, Roskilde, Denmark) on modified TYI-S-33 medium as previously described (Clark and Diamond, 2002). Subcultures were performed by inoculating 100 μl of cells from a confluent culture detached by incubation on ice for 15 min to a new tube containing 10 ml culture medium (Müller et al., 2006). Trophozoites were harvested by incubation on ice for 15 min followed by centrifugation (300×g, 10 min, 4°C).

### Transfection of *Giardia lamblia* Trophozoites

Transfection with the plasmid pPAC-V-GusA (Müller et al., 2009) and selection of transgenic trophozoites were performed as previously described (Yee and Nash, 1995; Singer et al., 1998). Briefly, prior to transfection, 1 µg of pPAC-V-GusA (in 20 µl digest mix) were linearized by digestion with SwaI (New England Biolabs, Ipswitch, MA) according to the instructions by the manufacturer. Then, 10^7^ trophozoites from a confluent culture were mixed with the digested plasmid DNA and incubated on ice for 5 min. Electroporation of trophozoites with linearized plasmid DNA was done in a 0.4 cm cuvette using an ECM 600 (BTX, San Diego, CA) at setting 350 V, 1000 µF, and 720 Ω. Electroporated trophozoites were immediately transferred to 10 ml medium in a plastic tube and incubated overnight at 37°C before puromycin was added to a final concentration of 100 µM. After 4 days, the medium was replaced by fresh medium containing 100 µM puromycin and drug-resistant cells visible after about 8 days post transfection were grown to confluence and then passaged once in the same medium. Before shotgun mass spectrometry analysis, the untransfected cultures were routinely passaged two times, the GusA-transfected cultures in absence of puromycin. Pellets were washed three times with ice-cold PBS, counted, and stored at −80°C for subsequent proteomic analysis or for enzymatic assays, respectively.

### Proteomics

Cell pellets were lysed in 100 μL 8M urea/100 mM Tris/HCl pH8/cOmplete™ protease inhibitor cocktail (Roche Diagnostics, Rotkreuz, Switzerland) by incubation for 15 min at room temperature followed by 15 min in an ultrasonic water bath. Protein concentration was determined by BCA assay, followed by reduction and alkylation of proteins with 10 mM DTT for 30 min at 37°C and 50 mM iodoacetamide for 30 min at 37°C in the dark. Proteins were precipitated at −20°C by addition of 5 vol cold acetone and incubation at −20°C overnight. All liquid was carefully removed and the pellet dried in ambient air for 15 min before reconstitution of proteins to a concentration of 1mg/ml in 8 M urea, 50 mM Tris-HCl pH 8.0. An aliquot corresponding to 10 μg protein was digested by trypsin (1:50 trypsin/protein ratio) for 6 hours at 37°C after dilution of urea concentration to 1.6M with 20 mM Tris-HCl pH 8.0 and 2 mM CaCl_2_. The digests were acidified with TFA (1%) and analyzed by LC-MS/MS. Three repetitive injections of an aliquot corresponding to 500 ng protein digest were analyzed on an EASY-nLC 1000 coupled to a QExactive HF mass spectrometer (ThermoFisher, Reinach, Switzerland). Peptides were trapped on an Acclaim PepMap100 C18 pre-column (3μm, 100 Å, 300 μm x 5 mm, ThermoFisher, Reinach, Switzerland) and separated by backflush on a C18 column (3μm, 100 Å, 75μm x 15 cm, Nikkyo Technos, Tokyo, Japan) by applying a 60 min gradient of 5% acetonitrile to 40% in water, 0.1% formic acid, at a flow rate of 400 nl/min. Peptides of m/z 400–1,400 were detected with resolution of 60,000 applying an automatic gain control (AGC) target of 1E06 and a maximum ion injection time of 50 ms. A top fifteen data dependent method for precursor ion fragmentation with a stepped 27% normalized collision energy was applied with the following settings: precursor isolation width of 1.6 m/z, resolution 15,000, AGC of 1E05 with a minimum target of 1E03, maximum ion time of 110 ms, charge exclusion of unassigned and 1+ ions, peptide match on, and dynamic exclusion for 20 s, respectively. The mass spectrometry proteomics data have been deposited to the ProteomeXchange Consortium *via* the PRIDE (Perez-Riverol et al., 2019) partner repository with the dataset identifier PXD022565.

### Statistics

The MS data for each strain consisted of three biological replicates, with three technical replicates each. All MS data were processed by MaxQuant (version 1.6.14.0) with matching between runs for the same strain activated, but not between different strains, in order to avoid over-interpretation of the data. Fragment spectra were interpreted against a recent *Giardia* protein sequence database in FASTA format (GiardiaDB-47_GintestinalisAssemblageAWB_AnnotatedProteins), supplemented by the two protein sequences of GusA and Pac from Swissprot database (accession numbers P05804 and P13249). The trypsin cleavage rule allowed amide bond cleavage after lysine and arginine but not if a proline follows and up to three missed cleavage sites, fixed carbamidomethylation modification of cysteine residues, variable oxidation of methionine and acetylation of protein N-termini. Precursor and fragment mass tolerances were set to 10 and 20 ppm, respectively. Peptide spectrum matches, peptide and protein group identifications were filtered to a 1% false discovery rate (FDR) based on reversed database sequence matches, and a minimum of two razor or unique peptides were required to accept a protein group identification.

Protein identifications considered as contaminations (e.g. trypsin) as well as proteins identified only by site were removed for statistical validation. The normalized label-free quantification (LFQ) protein group intensities as calculated by MaxQuant and a top3 approach were used for relative proteome quantifications. For top3, peptide intensities were median-normalized to the global median, then missing peptide imputation was done per sample by drawing values from a Gaussian distribution of width 0.3x sample standard deviation centered at the sample distribution mean minus 1.8x sample standard deviation if there were at least two peptide intensities in a group, otherwise a Maximum Likelihood Estimation (MLE) method was applied, followed by summation of the three most intense peptides per protein group to a surrogate of the real protein abundance, named iTop3. For LFQ values, we imputed with the same left-censored method as for peptides if there was a single missing protein LFQ value in the three biological replicates. Any remaining missing values were again imputed by the MLE method. In order to perform statistical tests, missing iTop3 or LFQ values were further imputed at the protein level using the MLE method, and the final imputed LFQ or iTop3 values were called iLFQ or iiTop3, respectively.

Differential expression tests were performed by applying the (Welch-) Student’s t-test (unequal variance). Log2-fold changes and adjusted p-values (FDR-controlled Benjamini and Hochberg correction) were reported. Furthermore, a check was performed by repeating the imputation cycle and significance testing 20 times. Protein groups with a persistent reporting of differential expression were considered as true differentially expressed between the two groups. A log2-fold change of at least one and a corrected p-value of ≦0.05 were required to be considered as significant. Statistical testing and imputation were made using a set of freely available R package tools running under R studio.

## Results

### Proteome Parameters of *Giardia lamblia* Trophozoites

**Figure 1:**
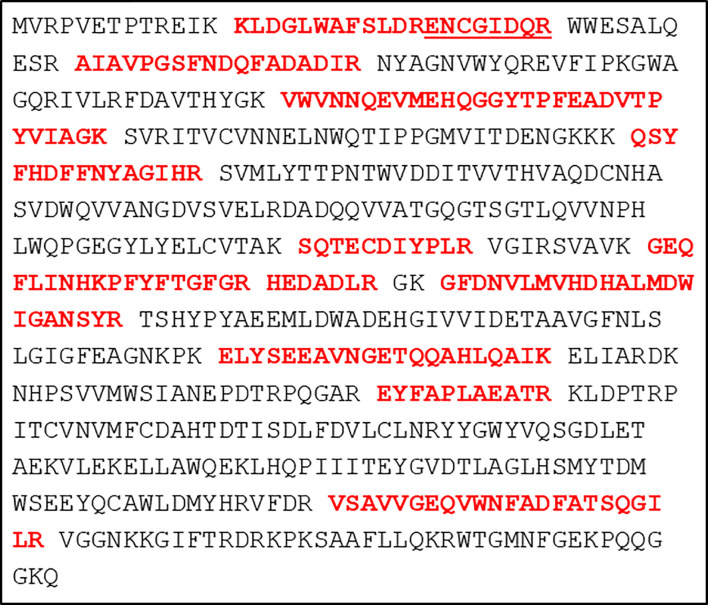
Primary sequence of the transgene *E. coli* glucuronidase A with the identified peptides highlighted in bold red letters. The underlined sequence is a separate peptide generated from the longer peptide sequence with a missed trypsin cleavage site at position twelve. Otherwise, trypsin cleavage sites are indicated by a space. Each line consists of 40 amino acids.

Shotgun mass spectrometry of the proteomes of untransfected *G. lamblia* WBC6 trophozoites (WT) and of trophozoites transfected with a plasmid containing the *S. alboniger* puromycin-N-acetlytransferase (Pac) as a resistance marker and the *E. coli* glucuronidase A gene (Gus A) resulted in the identification of 20’091 unique peptides matching to 1’705 proteins. Moreover, 12 unique peptides matching to GusA were consistently detected in the transfected trophozoites, but not in the WT. The identified peptides are depicted in **Figure 1**. Unique peptides matching to Pac could not be detected.

The expression levels of GusA in the transfected trophozoites were compared to four housekeeping proteins expressed at equal LFQ and iTop3 levels in both transfected and untransfected strains, namely pyruvate-ferredoxin oxidoreductase, arginine deiminase, thioredoxin reductase, and glutamate dehydrogenase. The levels of GusA were one to two magnitudes (in base 10) lower than the levels of these proteins (**Table 1**).

**Table 1:** Major housekeeping proteins and transgenes in untransfected *Giardia lamblia* WBC6 wildtype (WT) and *Escherichia coli* glucuronidase A (GusA)-transfected trophozoites.

Protein	Accession No.	LFQ		iTop3	
		WT	GusA	WT	GusA
Pyruvate ferredoxin oxidoreductase	17063	10,464 ± 407	8,096 ± 70	249 ± 36	196 ± 17
Arginine deiminase	112103	31,263 ± 450	39,025 ± 1,129	1,110 ± 431	1,550 ± 289
Thioredoxin-reductase	9827	1,725 ± 556	1,804 ± 458	108 ± 42	144 ± 33
Glutamate dehydrogenase	21942	10,315 ± 1015	7,159 ± 826	309 ± 39	216 ± 39
Glucuronidase A	P05804	nd	128 ± 7	nd	13 ± 2
Puromycin-N acetyltransferase	P13249	nd	nd	nd	Nd

Mean values (± SD) of LFQ, and iTop3 levels (x10^6^) are given for three biological replicates. The accession numbers of the transgenes are from the Uniprot database, the others from GiardiaDB. nd, not detected.

### Transfection Induces Significant Changes in *Giardia lamblia* Trophozoite Proteomes

**Figure 2:**
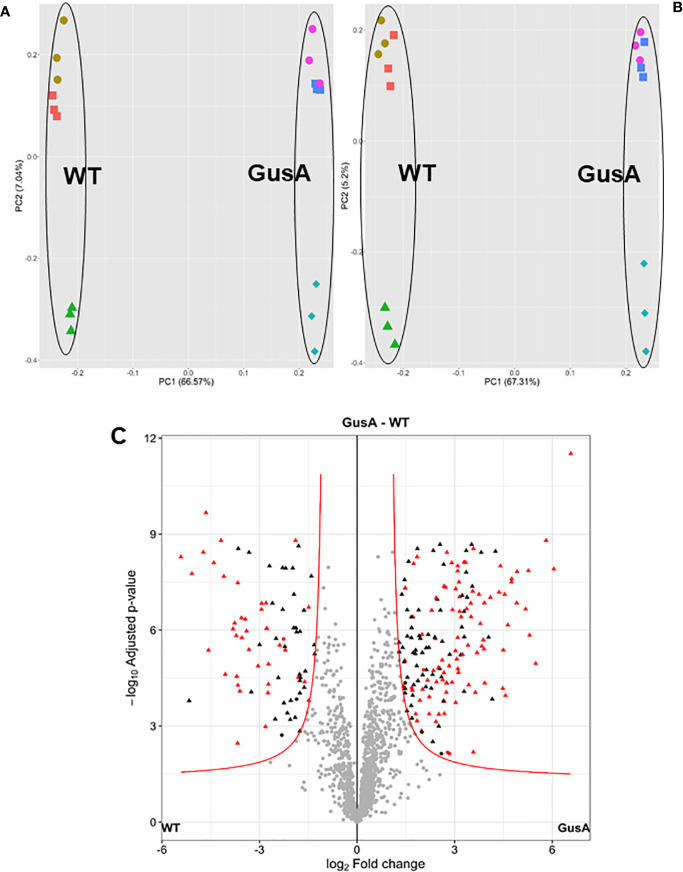
Principal component analysis plots **(A, B)** and volcano plot **(C)** of proteome data set from *G. lamblia* trophozoites transfected with a plasmid containing *E. coli* glucuronidase A (GusA) and of not transfected, control trophozoites (WT). The principal component analysis plots show for each strain, all technical and biological (red square, brown circle, green triangle for WT; pink circle, blue square, turquoise diamond for GusA) replicates based on iiTop3 data **(A)** or iLFQ data **(B)**. The volcano plot **(C)** is based on iiTop3 data. Differential proteins identified by both iiTop3 and iLFQ algorithms are depicted in red.

Overall analysis of the data by principal component analysis (PCA) revealed that the proteomes from WT and GusA trophozoites were distinctly separated along the principal component 1. As expected, technical replicates clustered together, whereas biological replicates were separated by principal component 2 for both groups (**Figures 2A, B**). Differences in the proteome patterns of both strains were confirmed by Volcano plot analysis (**Figure 2 C**).

### Differentially Expressed Proteins

**Figure 3:**
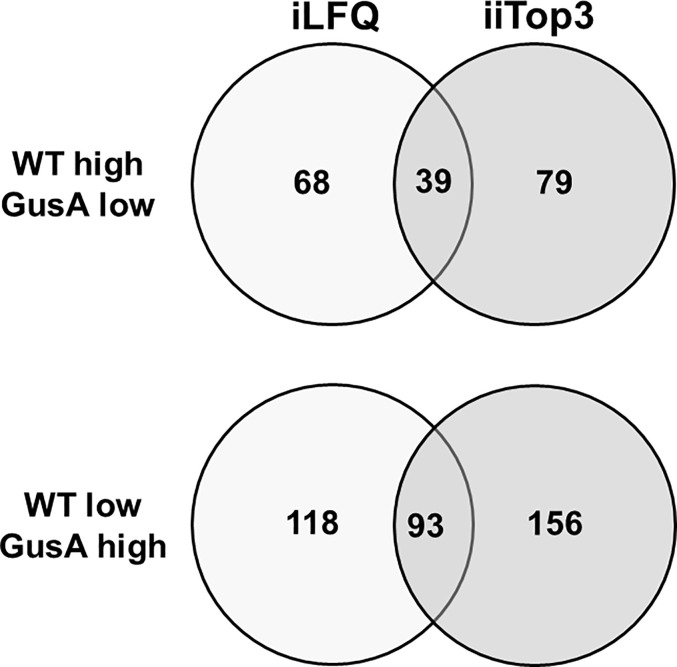
Venn diagram depicting the number of differentially expressed proteins in transfected and untransfected *G. lamblia* trophozoites. Untransfected trophozoites (WT) and trophozoites transfected with *E. coli* glucuronidase A were subjected to MS shotgun analysis as described in Materials and Methods. The differential proteins were determined *via* the iLFQ, the iiTop3 algorithm or *via* both.

Further analysis of the dataset revealed that the proteomes of WT and GusA contained 132 differentially expressed proteins, namely 39 with higher levels in WT and 93 with higher levels in GusA trophozoites, when the evaluation was done by both iLFQ and iiTop3 algorithms as shown in **Figure 3**.

Besides 70 differentially expressed (DE) hypothetical proteins, the two categories with the highest numbers of DE proteins, namely 17, comprised surface antigens and proteins involved in intermediary metabolism, followed by 14 proteins involved in gene expression, cell cycle and development and 14 proteins involved in cytoskeleton, flagella, adhesion and organelle transport (**Table 2**). The complete list of the accession numbers of the DE proteins is given as **Supplementary Table S1**.

**Table 2:** Overview of differentially expressed proteins in untransfected *G. lamblia* wildtype (WT) and *E. coli* glucuronidase A-transfected trophozoites (GusA).

(Hypothetical) function	Higher in WT	Higher in GusA
Surface antigens: (a) Variant-specific surface proteins	1	14
(b) High cysteine membrane proteins	2	0
21.1 proteins (ankyrin repeat proteins without kinase domain)	1	3
Gene expression, cell cycle, development	3	11
Intermediary metabolism	5	12
Transport of micromolecules	0	1
Cytoskeleton, flagella, adhesion and organelle transport	2	5
Chaperones	0	2
Hypothetical	25	45
Total	39	93

The proteins were identified by MS shotgun. Only proteins, which were differential by both iiTop3 and iLFQ algorithms were considered. The complete dataset is available online, the GiardiaDB open reading frame numbers and annotations of the differentials as **Supplementary Table S4**.

### Antigenic Variation

**Figure 4:**
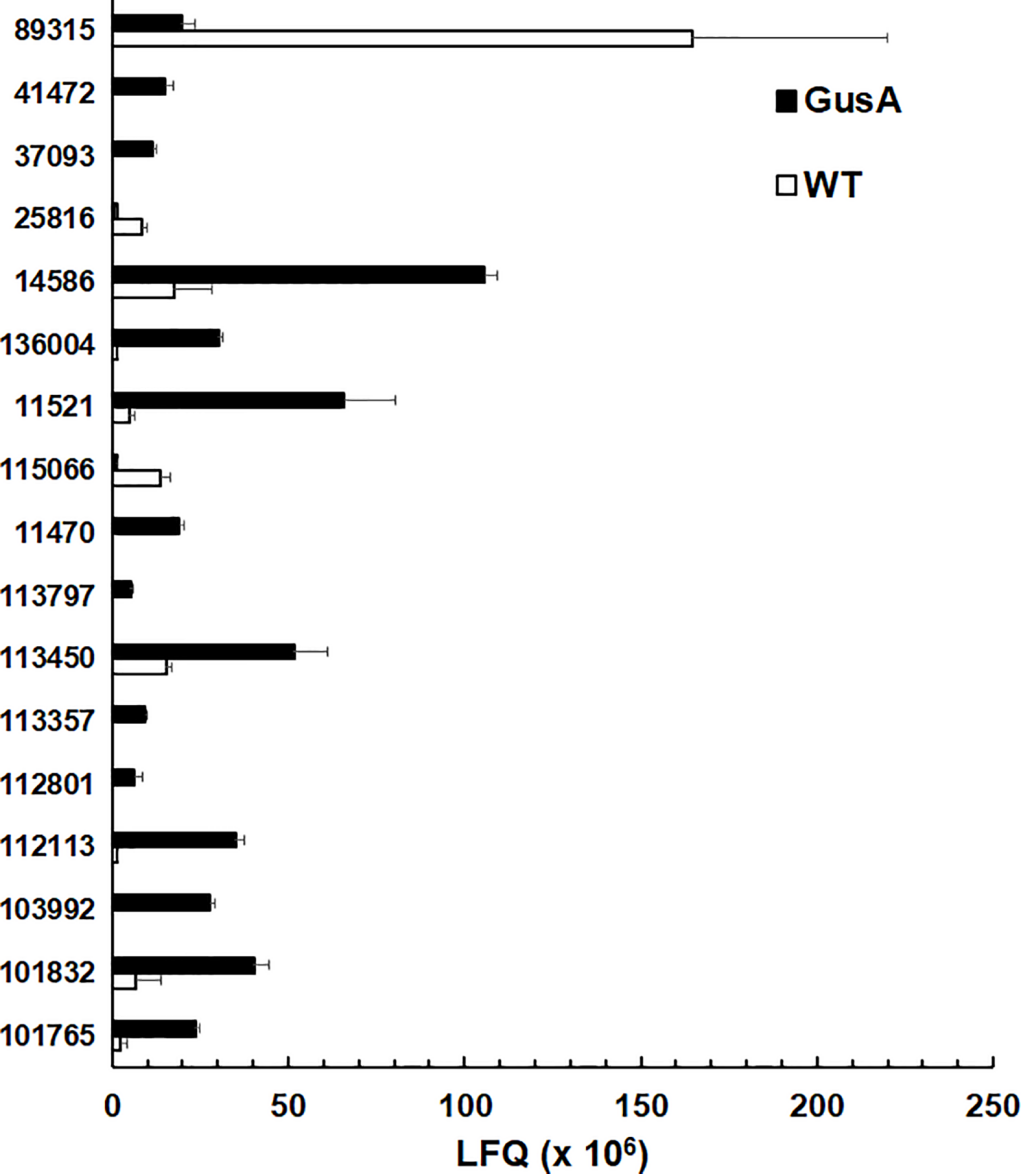
Quantitative assessments of differential surface antigens. Untransfected *G. lamblia* WBC6 trophozoites (WT; white bars) and *E. coli* glucuronidase A-transfected trophozoites (GusA; black bars) were subjected to MS shotgun analysis as described in *Materials and Methods* section. For all proteins, mean values ± one standard deviation for LFQ intensities (x10^6^) in three biological replicates are shown. The proteins are termed by their respective accession numbers in the GiardiaDB. 25816 and 113416 are high cysteine membrane proteins.

As mentioned above, surface antigens constituted one of the two categories with the highest amount of DE proteins, namely 17. In particular, 14 variant-specific surface proteins (VSP) had significantly higher levels in GusA-transfected trophozoites and one VSP in wildtype trophozoites. Conversely, only two high cysteine membrane proteins (HCMP), namely 115066 and 25816, had significantly higher levels in WT than in GusA trophozoites. The VSP 89315 was the predominant differential surface antigen with significantly higher levels in wildtype trophozoites. VSP14586 was the predominant DE VSP in GusA trophozoites (**Figure 4**).

The expression levels of the major surface antigens, namely the VSPs 188, 88, 8, and the HCMP with the ORF number 15317, however, were not significantly affected (**Table 3**).

**Table 3:** Major surface antigens without significant differences in expression levels between untransfected *G. lamblia* WBC6 wildtype (WT) and *E. coli* glucuronidase A (GusA)-transfected trophozoites.

Accession N°	Annotation	LFQ		iTop3	
		WT	GusA	WT	GusA
GL50803_101074	VSP with INR; VSP-88	1,315 ± 172	1,740 ± 148	746 ± 162	975 ± 185
GL50803_112207	VSP	102 ± 44	35 ± 2	168 ± 88	24 ± 2
GL50803_137612	VSP	195 ± 28	352 ± 27	119 ± 10	218 ± 24
GL50803_137613	VSP with INR; VSP-188	3,368 ± 217	3,170 ± 96	3,104 ± 96	2,728 ± 237
GL50803_137617	VSP	102 ± 3	123 ± 4	66 ± 3	79 ± 3
GL50803_137618	VSP with INR; VSP-8	979 ± 173	637 ± 54	619 ± 157	386 ± 37
GL50803_15317	HCMP group 1	835 ± 110	401 ± 23	495 ± 102	330 ± 29
GL50803_221693	(hypothetical), VSPA6	124 ± 60	48 ± 4	179 ± 75	42 ± 3
GL50803_33279	VSP	181 ± 12	118 ± 9	276 ± 6	150 ± 4

Mean values (± SD) of LFQ and iTop3 levels (x10^6^) are given for three biological replicates. Only surface antigens with LFQ levels above 10^8^ in at least one strain are shown. The accession numbers of the GiardiaDB are given. HCMP, high cysteine membrane protein; VSP, variant-specific surface protein.

## Discussion

The present dataset confirms our initial hypothesis that the transfection of *G. lamblia* by plasmids followed by an antibiotic-based selection of transfected trophozoites affects the whole genome expression pattern thereby confirming and extending previous findings (Su et al., 2007). Furthermore, it induces antigenic variation like other drug-based selections (Emery et al., 2018; Müller et al., 2019) or in strains from different genetic backgrounds (Emery et al., 2014; Emery et al., 2015a; Müller et al., 2020a). For instance, we have found 17 DE surface antigens between WT and Gus (one permutation) and 89 between WT and nitro drug resistant C4 cells grown either in the presence of metronidazole or nitazoxanide (three permutations), as shown in a previous study (Müller et al., 2019; in particular **Supplementary Table S2** therein).

Since antigenic variation is caused by epigenetic (Lagunas-Rangel and Bermudez-Cruz, 2018) and post-transcriptional (Prucca et al., 2008) mechanisms, the degree of variation could be used as a tool to estimate the impact of a given treatment, in our case transfection, on these mechanisms. As generally admitted, trophozoites express only one VSP on their surface at the same time (Nash et al., 2001). Thus, the fact that the number of differentially expressed VSPs is higher in the transfected than in the untransfected trophozoite population is indicative for a higher degree of diversification most likely caused by the selection process. Moreover, the transfection and the subsequent selection procedure may influence metabolism, cell organization and motility, as indicated by a number of differentials in these categories. In fact, we observe only small metabolic differences between WT and GusA transfected strains, as published in a previous study (Müller et al., 2020b).

It is surprising that 12 unique peptides can be attributed to the GusA protein thereby allowing an easy identification, but no peptides to the puromycin resistance marker Pac protein, which has allowed to select the transfectants. The expression of both proteins is controlled by the same promoter, namely the strong promoter of the glutamate dehydrogenase (Singer et al., 1998). The lack of unique Pac peptides could be due to a high similarity to peptides derived from *Giardia* proteins—what is, however, very unlikely—or to a rapid post-transcriptional silencing.

Consequently, each experiment investigating the role of a given transgene should contain an irrelevant transfection (e.g. with GusA) as a control. Untransfected WT trophozoites do not reflect the impact of the transfection and selection procedures on gene expression. So far, it is unclear how other genetic manipulations such as RNA virus-mediated transfection (Janssen et al., 2015) or CRISPR/Cas9 (Lin et al., 2019; McInally et al., 2019) affect gene expression in *G. lamblia*. Proteome studies should be performed with trophozoites transfected by these methods and investigate the impact on epigenetic and post-transcriptional regulation of gene expression by estimating the degree of antigenic variation. Further, more detailed studies comparing either different selection methods, or different control genes, or both, would be useful for helping researchers to determine methods that induce minimal proteomic perturbation. Indeed, the ideal vector control will induce minimal proteomic changes relative to wild-type untransfected cells. By including a reference transgene such as Gus A, expressing a clearly detectable, unique peptide may help to establish an internal standard, e.g. for comparative studies in different genetic backgrounds.

## Data Availability Statement

The mass spectrometry proteomics data have been deposited to the ProteomeXchange Consortium via the PRIDE (Perez-Riverol et al., 2019) partner repository with the dataset identifier PXD022565.

## Author Contributions

MH and SB analyzed the trophozoite proteomes. NM and JM conceptualized the study, constructed and cultured the strains, and evaluated the results. JM wrote the manuscript. All authors contributed to the article and approved the submitted version.

## Funding

This research was funded by the Swiss National Science Foundation, grant number 31003A_163230 and grant no. 206021-128736.2.

## Conflict of Interest

The authors declare that the research was conducted in the absence of any commercial or financial relationships that could be construed as a potential conflict of interest.
