# Supplementary material for: Transfection With Plasmid Causing Stable Expression of a Foreign Gene Affects General Proteome Pattern in Giardia lamblia Trophozoites
Source: Front Cell Infect Microbiol. 2020 Dec 18;10:602756. doi: 10.3389/fcimb.2020.602756 (PMC7775365; doi:10.3389/fcimb.2020.602756)
Supplement: Supplementary file 1 [file Table_1.docx]

Supplementary Material

# Supplementary Data

The entire shotgun MS dataset is available online (MS_dataset; web address to be added).

# Supplementary Table

**Table S1**. List of proteins with significantly different expression levels in *G. lamblia* WBC6 trophozoites (WT) and *E. coli* glucuronidase A-transfected trophozoites (GusA). The respective accession numbers and annotations in the GiardiaDB are given.

| **Higher in WT** | | **Higher in GusA** | |
| --- | --- | --- | --- |
| **Accession N°** | **Annotation** | **Accession N°** | **Annotation** |
| GL50803_102455 | Kinesin-6 | GL50803_101168 | Protein 21.1 |
| GL50803_103164 | H-SHIPPO 1 | GL50803_10161 | hypothetical protein |
| GL50803_10522 | hypothetical protein | GL50803_101765 | VSP |
| GL50803_113788 | hypothetical protein | GL50803_101832 | High cysteine protein (VSP4A1) |
| GL50803_115066 | High cysteine membrane protein VSP-like | GL50803_102248 | Coiled-coil protein |
| GL50803_13616 | Glycine-rich protein | GL50803_103838 | Kinase |
| GL50803_13945 | hypothetical protein | GL50803_103888 | hypothetical protein |
| GL50803_14098 | ATP-dependent RNA helicase | GL50803_103992 | VSP |
| GL50803_14507 | hypothetical protein | GL50803_10569 | hypothetical protein |
| GL50803_14967 | hypothetical protein | GL50803_10679 | hypothetical protein |
| GL50803_15139 | hypothetical protein | GL50803_10765 | MCM7 |
| GL50803_15472 | hypothetical protein | GL50803_11040 | Kinase |
| GL50803_16269 | FAD synthetase | GL50803_112113 | VSP |
| GL50803_17043 | Glyceraldehyde 3-phosphate dehydrogenase | GL50803_112408 | hypothetical protein |
| GL50803_17531 | hypothetical protein | GL50803_112801 | VSP |
| GL50803_22385 | Protein 21.1 | GL50803_113357 | VSP |
| GL50803_25035 | Protein tyrosine phosphatase | GL50803_113450 | VSP with INR |
| GL50803_25816 | High cysteine membrane protein Group 1 | GL50803_113797 | VSP with INR |
| GL50803_2732 | Hypothetical protein | GL50803_114674 | Hypothetical protein |
| GL50803_2796 | Kinase | GL50803_11470 | VSP with INR |
| GL50803_2864 | hypothetical protein | GL50803_11497 | hypothetical protein |
| GL50803_30233 | hypothetical protein | GL50803_11521 | VSP |
| GL50803_33030 | hypothetical protein | GL50803_11566 | hypothetical protein |
| GL50803_41619 | hypothetical protein | GL50803_12215 | S-adenosylmethionine-dep. methyltransferase |
| GL50803_4323 | hypothetical protein | GL50803_136004 | VSP |
| GL50803_4369 | Dual specificity phosphatase Cdc25 | GL50803_13809 | hypothetical protein |
| GL50803_4587 | hypothetical protein | GL50803_13988 | hypothetical protein |
| GL50803_4653 | Serpin 1 | GL50803_14216 | Kinase |
| GL50803_7018 | hypothetical protein | GL50803_14539 | Protein 21.1 |
| GL50803_7706 | hypothetical protein | GL50803_14573 | Tenascin-X |
| GL50803_7800 | hypothetical protein | GL50803_14586 | VSP with INR |
| GL50803_7865 | L-asparaginase | GL50803_14602 | hypothetical protein |
| GL50803_8058 | hypothetical protein | GL50803_14843 | Chaperone protein dnaJ |
| GL50803_8616 | hypothetical protein | GL50803_14906 | hypothetical protein |
| GL50803_8725 | hypothetical protein | GL50803_14940 | hypothetical protein |
| GL50803_87261 | hypothetical protein | GL50803_14997 | hypothetical protein |
| GL50803_89315 | VSP | GL50803_15204 | ERP3 |
| GL50803_90665 | hypothetical protein | GL50803_15515 | hypothetical protein |
| GL50803_91476 | hypothetical protein | GL50803_15930 | RNA helicase |
|  |  | GL50803_15958 | Ser/Thr protein kinase |
|  |  | GL50803_15987 | hypothetical protein |
|  |  | GL50803_16157 | hypothetical protein |
|  |  | GL50803_16199 | Coiled-coil protein |
|  |  | GL50803_16230 | Hypothetical protein |
|  |  | GL50803_16415 | hypothetical protein |
|  |  | GL50803_16460 | Kinase |
|  |  | GL50803_16484 | hypothetical protein |
|  |  | GL50803_16534 | Protein 21.1 |
|  |  | GL50803_16944 | hypothetical protein |
|  |  | GL50803_16985 | hypothetical protein |
|  |  | GL50803_16986 | Kinase |
|  |  | GL50803_17025 | Nuclear ATP/GTP-binding protein |
|  |  | GL50803_17123 | hypothetical protein |
|  |  | GL50803_17198 | Leucine-rich repeat protein |
|  |  | GL50803_17283 | hypothetical protein |
|  |  | GL50803_17389 | SUA5 protein |
|  |  | GL50803_17412 | hypothetical protein |
|  |  | GL50803_17574 | Coiled-coil protein |
|  |  | GL50803_1770 | DRL1 protein |
|  |  | GL50803_2082 | Kinase |
|  |  | GL50803_21110 | hypothetical protein |
|  |  | GL50803_23357 | NYD-SP28 protein |
|  |  | GL50803_23447 | hypothetical protein |
|  |  | GL50803_23767 | hypothetical protein |
|  |  | GL50803_24453 | hypothetical protein |
|  |  | GL50803_32571 | hypothetical protein |
|  |  | GL50803_32681 | hypothetical protein |
|  |  | GL50803_33989 | hypothetical protein |
|  |  | GL50803_3491 | hypothetical protein |
|  |  | GL50803_37093 | VSP |
|  |  | GL50803_3920 | hypothetical protein |
|  |  | GL50803_4017 | hypothetical protein |
|  |  | GL50803_40244 | P24 |
|  |  | GL50803_41472 | VSP |
|  |  | GL50803_4192 | ARL2 |
|  |  | GL50803_4711 | hypothetical protein |
|  |  | GL50803_5374 | Tubulin specific chaperone B |
|  |  | GL50803_5949 | Tetratricopeptide repeat protein |
|  |  | GL50803_7126 | hypothetical protein |
|  |  | GL50803_7747 | hypothetical protein |
|  |  | GL50803_7797 | Alpha-5 giardin |
|  |  | GL50803_8140 | GTP-binding protein ARD-1 |
|  |  | GL50803_8157 | RNA polymerase II subunit Rpb5b |
|  |  | GL50803_8456 | Tubulin tyrosine ligase |
|  |  | GL50803_8464 | hypothetical protein |
|  |  | GL50803_86440 | Peptidyl-tRNA hydrolase |
|  |  | GL50803_86934 | Kinase |
|  |  | GL50803_8708 | Eukaryotic translation initiation factor 1A |
|  |  | GL50803_8741 | Dipeptidyl-peptidase I precursor |
|  |  | GL50803_9077 | Inositol 5-phosphatase 4 |
|  |  | GL50803_9134 | hypothetical protein |
|  |  | GL50803_91911 | Translation initiation factor eIF-2B alpha SU |
|  |  | GL50803_9741 | ABC transporter |

**
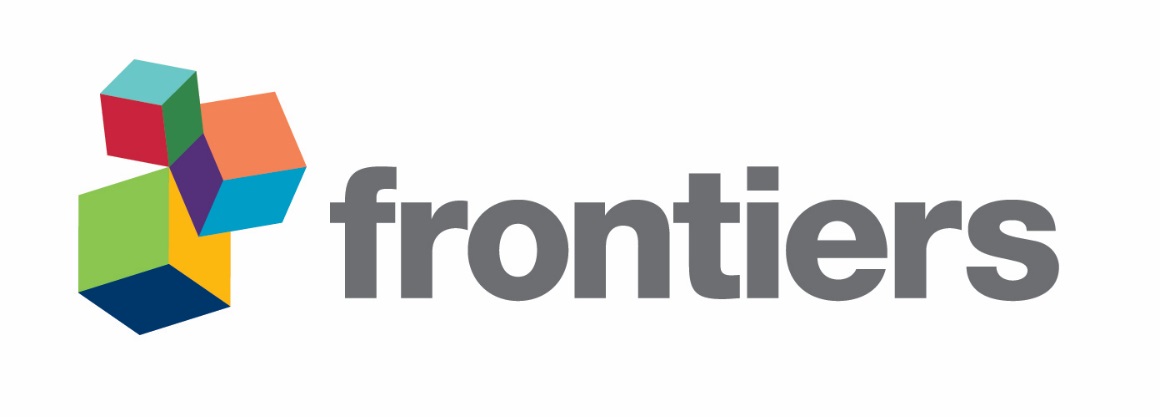
**
